# Psychiatric emergencies and trauma : the impact of stress in emergency nurses

**DOI:** 10.1192/j.eurpsy.2024.1176

**Published:** 2024-08-27

**Authors:** N. Baabouchi

**Affiliations:** psychatry, arrazi hospital, rabat, Morocco

## Abstract

**Introduction:**

Mental health at work is increasingly an essential element to assess, especially in sectors with a high risk of psychological and physical stress. Working in a healthcare environment and particularly work in a psychiatric environment can constitute a psychological risk for workers. Among the risks faced by emergency psychiatric medical staff is the risk of developing PTSD (post traumatic stress disorder), which occurs after a traumatic event and results in moral suffering and physical complications that profoundly alter life:personal, social and professional life.

**Objectives:**

Screening psychiatric emergency nurses for post-traumatic stress disorders.

**Methods:**

This is a cross-sectional study carried out in the psychiatric emergency department of the Arrazi University Hospital in Salé, using an anonymous questionnaire distributed to nurses. It includes a 1st part on sociodemographic and professional data, a 2nd part focused on the evaluation of mental health through the GHQ12 and a 3rd part which evaluates post-traumatic stress made by the scale of post-traumatic stress disorder (PCLS).

**Results:**

60 pourcent of women are more able to have ptsd disorder

40 pourcent men 95 pourcent are under the age of 30 and 5 pourcent have more than 30 years

80 pourcent have morked less than 5 years in the emergency hospital

and 20 pourcent have worked more 73 pourcent have scored more than 44 in pcls score

23 pourcent have scored less than 44 in pcls score

**Conclusions:**

This work highlighted an extremely high rate of exposure to a violent event among psychiatric emergency nurses, even if in this study the prevalence of PTSD found among nurses is lower than expected, in this professional environment overexposure a violence requires special attention to protect and prevent the development of PTSD in professionals

**Disclosure of Interest:**

None Declared

